# Study on the Influencing Factors of the Migration and Transformation Behavior of Hexavalent Chromium in a Soil–Groundwater System: A Review

**DOI:** 10.3390/toxics14010098

**Published:** 2026-01-21

**Authors:** Xiangyi Zhao, Mengqiuyue Hao, Tuantuan Fan, Ang Liu, Chenglian Feng

**Affiliations:** 1 State Key Laboratory of Environmental Criteria and Risk Assessment, Chinese Research Academy of Environmental Sciences, Beijing 100012, China; zhaoxiangyi24@mails.ucas.ac.cn (X.Z.); haomengqiuyue23@mails.ucas.ac.cn (M.H.); fantt7722@163.com (T.F.); ang_liuer@163.com (A.L.); 2 College of Water Science, Beijing Normal University, Beijing 100875, China

**Keywords:** chromium VI, soil-groundwater system, transport, transformation, influencing factors

## Abstract

The migration and transformation of Cr(VI) are primarily regulated by soil minerals, soil flora and fauna, hydrological conditions, and microbial communities, with these mechanisms being influenced by pH, temperature, and oxygen levels. In terms of single environmental media, relatively extensive research has been conducted on the behaviors of Cr(VI). However, studies on the migration and transformation of Cr(VI) from the perspective of the soil–groundwater multimedia system are rarely published. Therefore, this study comprehensively analyzes the migration and transformation behaviors of Cr(VI) from the perspective of the entire soil–groundwater system. By synthesizing the effects of individual factors, such as pH and organic matter, on Cr(VI) in both soil and groundwater, as well as interactions among these factors, we systematically clarify the patterns governing Cr(VI) migration and transformation under multi-factor coupling. Through the analysis of multiple factors in the complex system, the redox fluctuation zone at the soil–groundwater interface is a hot spot for Cr(VI) transformation, and the synergistic effect among climatic conditions, microbial community structure, and the aquifer interface significantly affects the transport efficiency of Cr(VI). The results of the present study could provide a theoretical framework for future research on the environmental behavioral effects of Cr(VI) at the soil–groundwater interface. Moreover, this study could provide important theoretical bases for the prevention and control of heavy metal pollution.

## 1. Introduction

The soil–groundwater system is a critical component of the ecosystem, closely linked to the foundation of human survival and ecological security, and thus holds significant research importance. Current studies, however, predominantly focus on either soil or groundwater as separate media, with limited attention to their synergistic interactions [1]. From a resource perspective, groundwater serves as a vital source of drinking water in arid and semi-arid regions, while soil supports agricultural production. Moreover, the soil–groundwater system acts as a major pathway for contaminant migration and transformation, characterized by attributes such as hidden pathways, hysteresis, and irreversibility [2,3]. Pollutants can infiltrate into groundwater through leaching and subsequently spread. Once contaminated, the system is hard to recover and costly to remediate [4]. Therefore, in-depth research on hydrogeochemical processes is essential, including adsorption–desorption, redox reactions, and microbial transformations. Such research will provide a scientific basis for precise remediation and sustainable management, supporting the restoration of soil and water ecosystems [5,6]. This work contributes to the vision of harmonious coexistence between humans and nature—a core goal of sustainable development.

**Figure 1:**
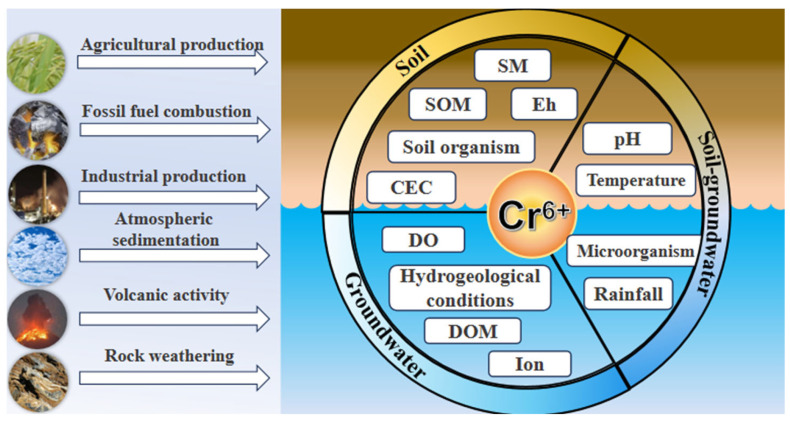
Sources and effects of Cr(VI) in soil and groundwater systems.

Among various pollutants, chromium is a typical heavy metal contaminant. It exists primarily in two valence states in the natural environment: hexavalent chromium (Cr(VI)) and trivalent chromium (Cr(III)) [7]. Cr(VI) exhibits high solubility, strong mobility, and high toxicity. It commonly exists as an oxyanion and can accumulate in the liver, kidneys, heart, blood, and endocrine glands through ingestion, dermal contact, or inhalation, leading to adverse hepatic effects and increased cancer risk. In contrast, Cr(III) occurs mainly in the form of chromite and is far less toxic. Therefore, reducing Cr(VI) to Cr(III) represents an effective strategy for decreasing chromium toxicity [8,9]. The pathways through which Cr(VI) enters the soil–groundwater environment are multidimensional, encompassing both anthropogenic and natural sources (Figure 1). According to [10], 68.95% of chromium contamination in groundwater from 1999 to 2022 was attributed to human activities. Among the sites affected, 56.43% had hexavalent chromium concentrations within the range of 0–10 mg/L. However, at sites with naturally occurring high concentrations of hexavalent chromium in groundwater, 75.00% exhibited levels below 0.2 mg/L. Wu et al. [11] collected data on chromium content from 1331 soil samples, with an average value of 85.01 mg/kg, which is significantly higher than the background level. Due to this dual nature, investigating its toxic mechanisms and transport behavior mechanism is crucial for managing typical heavy metal contamination, preventing ecological risks, promoting green industrial transition, and enabling safe reuse of chromium-contaminated sites [12].

Furthermore, current research remains largely focused on single-medium studies, and a systematic understanding of the kinetic mechanisms governing interfacial migration is still lacking. Therefore, this study focuses on research related to the transport and transformation of Cr(VI) in contaminated soils and groundwater. By searching databases such as Web of Science, Google Scholar, and CNKI, using core keywords including “soil,” “groundwater,” and “hexavalent chromium,” we systematically analyze and discuss the various factors influencing the transport and transformation patterns of Cr(VI) in the soil–groundwater system. Furthermore, multiple environmental factors are integrated to address existing knowledge gaps in this field. The study aims to enhance understanding of the environmental behavior of Cr(VI) and the influence of contextual factors, thereby providing new insights and recommendations for the development of green, economical, and practical remediation technologies in the future.

## 2. Factors Affecting the Transport and Transformation of Chromium VI in Soil

**Figure 2:**
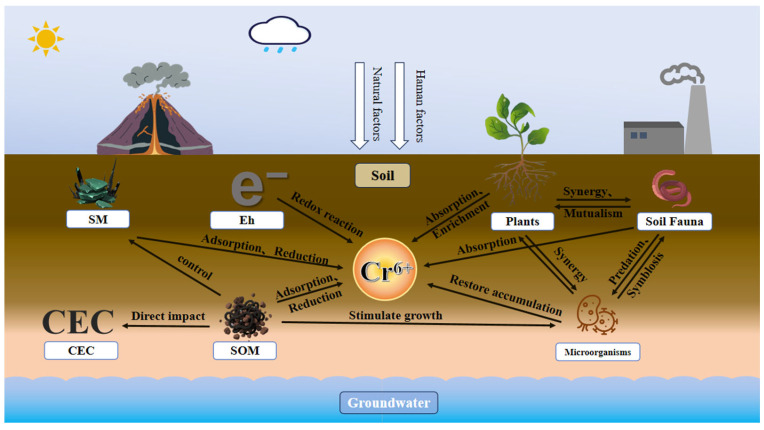
Schematic diagram of the influence of soil environmental factors on Cr(VI) migration and transformation.

The migration and transformation behaviors of hexavalent chromium (Cr(VI)) in soil systems are governed by interconnected factors categorized into two primary domains: soil physicochemical properties and biological activities. This chapter focuses primarily on the chemical properties of soil. Among chemical properties, soil pH and redox potential (Eh) constitute dominant regulators that directly dictate Cr(VI) speciation, solubility, adsorption dynamics, and redox transformation kinetics. Soil organic matter (SOM) exerts substantial control through electron donation, adsorption site provision, and microbial mediation, while cation exchange capacity (CEC) modulates adsorption site availability, and soil minerals influence Cr(VI) immobilization and migration via adsorption, catalysis, or direct reduction. Biological processes further regulate Cr(VI) dynamic balance. Microorganisms convert hexavalent chromium into trivalent chromium by participating in redox reactions, biosorption, and bioaccumulation processes [13]. Meanwhile, plant roots and soil fauna indirectly participate through absorption, exudate secretion, soil structural modification, and microhabitat alteration. These factors operate synergistically rather than in isolation, as physicochemical–biological interactions collectively governing Cr(VI) partitioning and transport within soil matrices (Figure 2).

### 2.1. Soil Physicochemical Properties

#### 2.1.1. pH

Soil pH is a critical property governing the partitioning and chemical speciation of metals in soil, thereby influencing their toxicity, mobility, and adsorption onto the solid-phase components [14]. The speciation of Cr(VI) is also regulated by soil pH: Cr(VI) primarily exists in anionic forms, such as CrO_4_^2−^ and Cr_2_O_7_^2−^ in soil solution. In the pH range of 6.5 to 14, CrO_4_^2−^ dominates; at pH 0.7 to 6.5, HCrO_4_^−^ and Cr_2_O_7_^2−^ prevail; below pH 0.7, H_2_CrO_4_ is the primary form [15].

Soil pH governs the migration and transformation of hexavalent chromium (Cr(VI)) in soil by regulating surface charge, redox state, and chemical speciation of chromium. Under acidic conditions, the soil surface carries a net positive charge, which effectively adsorbs Cr(VI) anions (e.g., HCrO_4_^−^), reduces their solubility in pore water, and thus restricts their mobility [16]. As pH increases, the negative charge on the soil surface increases, weakening the adsorption of Cr(VI) and enhancing its solubility and mobility. Simultaneously, pH strongly influences the stability of chromium valence states: the higher redox potential in acidic environments favors the reduction of Cr(VI) to Cr(III), while alkaline conditions tend to stabilize Cr(VI). Regarding chemical speciation, Cr(III) readily precipitates as Cr(OH)_3_ at pH > 6, resulting in low mobility, whereas Cr(VI) exists as highly soluble oxyanions (e.g., CrO_4_^2−^), exhibiting even greater solubility and mobility under alkaline conditions [14]. Furthermore, low pH generally promotes the desorption and dissolution of heavy metals, while high pH favors their precipitation. A highly significant negative correlation (r = −0.98, *p* < 0.01) is found between soil pH and Cr(VI) content, indicating that higher pH reduces Cr(VI) retention through altered metal ion interactions [17]. Li et al. [18] further confirmed pH as a pivotal factor controlling Cr(VI) transformation via shifts in surface charge and functional groups. At pH > 7, Cr(VI) mobility increases markedly. Additionally, pH influences microbial community activity, indirectly modulating Cr(VI) reduction kinetics. Overall, through these synergistic mechanisms, soil pH profoundly affects the adsorption–desorption, valence transformation, and solubility of Cr(VI), ultimately determining its environmental migration potential and associated risks.

#### 2.1.2. Redox Potential

Soil redox potential (Eh) reflects the relative abundance and activity of oxidized and reduced species, critically regulating Cr(VI) migration and transformation. Under oxidizing conditions (high Eh), Cr(III) oxidizes to Cr(VI), leading to an increase in Cr(VI) content in the soil [19]. Cr(VI) exists primarily in the form of chromate (CrO_4_^2−^) or dichromate (Cr_2_O_7_^2−^) ions, which are highly water-soluble and mobile. Conversely, under reducing conditions (low Eh), reductants such as Fe^2+^, S^2−^, and soil organic matter (SOM) donate electrons to reduce Cr(VI) to Cr(III), precipitating as low-solubility Cr(OH)_3_ and immobilizing Cr in surface soils. Wang et al. [20] observed that soil Cr(VI) concentration follows a logarithmic relationship with Eh, with peak migration in oxidizing soils (Eh = 320 mV) and diminished mobility as Eh decreases, accompanied by increased Cr(III) formation. It is found that the strong oxidizing condition (Eh = 400 mV) resulted in the greatest amount of Cr(VI) being leached and the highest effective diffusion coefficient, presumably due to oxidation of low mobile Cr (III) to high mobile Cr(VI) [21].

The influence of redox potential (Eh) on the environmental transport and transformation of hexavalent chromium (Cr(VI)) must be comprehensively evaluated in conjunction with chemical conditions, particularly pH. Collectively, in acidic media (pH < 6), the reduction kinetics of Cr(VI) are substantially enhanced due to elevated proton activity and favorable thermodynamic conditions. Conversely, under alkaline conditions (pH > 8), Cr(VI) exhibits significantly enhanced chemical stability owing to electrostatic repulsion from negatively charged soil colloids and reduced reduction propensity [22]. Simultaneously, Cr(III) readily precipitates as low-solubility Cr(OH)_3_, markedly improving its immobilization and reducing ecological risks. This pH-dependent speciation shift underscores the critical role of Eh-pH coupling in controlling chromium fate: acidic reducing environments maximize reduction rates but elevate dissolved Cr(III) mobility, while alkaline reducing conditions promote irreversible precipitation yet may form soluble hydroxo-complexes such as Cr(OH)_4_^−^ under specific Eh thresholds. Such mechanistic insights are essential for predicting chromium behavior in heterogeneous environmental matrices and designing targeted remediation strategies for contaminated sites. Specifically, in acidic and low-oxygen environments (pH < 6, Eh < 0), Cr(VI) reduction rates peak, but the resulting Cr(III) remains largely dissolved and highly mobile. In alkaline and low-oxygen conditions (pH > 8, Eh < 0), although Cr(VI) reduction can occur, Cr(III) often forms soluble hydroxyl complexes such as Cr(OH)_4_^−^, which enhance its mobility. In acidic and high-oxygen settings (pH < 6, Eh > 0), mineral surfaces strongly adsorb Cr(VI) anions. Nevertheless, the absence of effective reduction mechanisms means that both adsorbed and dissolved Cr(VI) remain mobile.

#### 2.1.3. Cation Exchange Capacity

Cation exchange capacity (CEC) quantifies the total exchangeable cations in soils and is a key factor influencing heavy metal speciation [23]. Soil CEC originates primarily from organic matter and increases with clay content, amorphous oxides, specific surface area, and nutrient levels. It directly reflects the abundance of available adsorption sites, including those on organic matter and metal hydroxides [24].

Low-CEC soils possess fewer negative surface charges, weakening adsorption of anionic Cr(VI) and facilitating leaching into groundwater. In contrast, high-CEC soils retain exchangeable cations such as Ca^2+^ Mg^2+^, which form “cation bridges” via electrostatic attraction to negatively charged colloids, indirectly enhancing Cr(VI) anion adsorption. It demonstrated that elevated CEC increases the availability of charged sites for Cr complexation, inhibiting Cr(VI) migration [25]. Qi et al. [17] further reported significant negative correlations between CEC and Cr(VI) content (r = −0.69, *p* < 0.05), acid-extractable Cr, and oxidizable Cr, indicating that higher CEC reduces chromium mobility and bioavailability by enhancing adsorption of anionic Cr(VI) onto soil solids.

The underlying mechanism lies in the enhancement of adsorptive fixation capacity for chromium particularly the anionic species Cr(VI) by soil solid-phase components due to elevated CEC [26]. Consequently, soils with high CEC reduce chromium solubility and mobility, thereby significantly mitigating its ecological and human health risks through immobilization.

#### 2.1.4. Soil Organic Matter

Soil organic matter (SOM) plays a multifaceted role in chromium dynamics. While less dominant than its role in hydrophobic organic pollutant partitioning, it still participates in the migration and transformation process of chromium through multiple pathways, and its mechanism of action is complex and cannot be ignored [27]. SOM participates in Cr migration through electron donation, adsorption, and microbial support. SOM acts as an electron donor to reduce Cr(VI) to less toxic Cr(III). Its surface functional groups such as carboxyl, carbonyl, and phenolic hydroxyl dissociate to generate negative charges, strong adsorption of Cr(III) cations and reducing mobility. SOM also supplies carbon and energy to Cr(VI)-reducing microbes, promoting bio-reducing reaction, which can indirectly regulate of chromium’s valence state transformation and distribution [28].

The reducing capacity of SOM varies with composition. It is found that water-soluble organic matter (WSOM) and hot-water-soluble organic matter (HWSOM) rich in microbial byproducts (MBPs) reduce Cr(VI) more effectively than humic acid (HA)-like components due to higher proportions of low-molecular-weight saturated compounds and CHO molecules [29]. Fresh, labile organic matter provides electrons for direct or microbial Cr(VI) reduction, while highly humified SOM excels at adsorbing and immobilizing Cr(III) [30]. Consequently, soil organic matter plays an important ecological role in alleviating the toxicity of chromium and its migration and transformation. It can not only act as an electron donor to directly reduce Cr(VI) to Cr(III), but also strongly adsorb and stabilize generated Cr(III) through its abundant functional groups. Additionally, it can indirectly promote the reduction of Cr(VI) by supporting microbial activity. However, the toxicity of Cr(VI) can lead to adverse effects such as deteriorated soil structure, reduced organic matter content, inhibited crop growth, and exacerbated soil erosion. The addition of organic amendments (e.g., animal manure, compost, biochar, etc.) can effectively improve the fertility of degraded soils by enhancing soil aggregation and stability. This approach is essentially a method of humifying organic matter, which subsequently strengthens the soil’s capacity to immobilize heavy metals. Specifically, compost can temporarily stabilize heavy metals through adsorption and complexation, but it is prone to decomposition and requires continuous application [31]. In contrast, biochar possesses a stable aromatic carbon structure and a high specific surface area, enabling the long-term immobilization of heavy metals such as Cr(VI) through mechanisms like adsorption, precipitation, complexation, and redox reactions, while also promoting soil carbon sequestration [32]. Therefore, increasing soil organic matter content represents a common and effective remediation technique for chromium-contaminated soil [33]. Nowadays, adding organic amendments such as compost, biochar, and plant residues is a common and effective in situ stabilization remediation technique in many practices for remediating chromium-contaminated soil both domestically and internationally [31,32].

#### 2.1.5. Soil Minerals

Soil minerals, which are the basic component of the soil inorganic compounds derived from rock weathering and pedogenesis, constitute the solid phase. They influence Cr(VI) migration through redox reactions, catalysis, and adsorption [34].

In redox and catalytic processes, manganese oxides play a key role. The oxidation capacity of MnO_2_ for Cr(III) increases with decreasing pH, as the reduction potential for the conversion of Cr(III) to Cr(VI) decreases under lower pH conditions. Iron-bearing minerals in nature can directly reduce Cr(VI) to Cr(III) and adsorb it onto their surfaces [35]. The reduction rate is influenced by environmental factors such as pH, temperature, and mineral composition. Acidity and alkalinity affect mineral solubility; for instance, the reduction of Cr(VI) by biotite increases with higher pH. Intermediate species such as Fe(II), Fe(III), and Mn(II), generated through surface adsorption or dissolution, can catalyze the reduction of Cr(VI) via electron transfer or ligand formation with other substances. Their catalytic effectiveness is positively correlated with the concentrations of Fe(II), Fe(III), and Mn(II). Additionally, environmental conditions such as temperature, pH, and light also affect the transformation of Cr(VI) by soil minerals [36].

In adsorption–desorption processes, soil exhibits a relatively weaker capacity for adsorbing and immobilizing Cr(VI) compared to Cr(III). The adsorption behavior is closely related to soil minerals and their surface charges. Soil minerals provide cationic sites on their surfaces, which offer abundant adsorption sites for anions such as CrO_4_^2−^ and Cr_2_O_7_^2−^, thereby enhancing the adsorption efficiency of Cr(VI) and reducing its mobility [37]. Dultz et al. [38] found that montmorillonite and vermiculite contain internal pores capable of retaining Cr(VI) through both surface binding and interlayer adsorption, with their adsorption capacities following a specific order and being influenced by conditions such as pH, temperature, ionic strength, and organic matter content. Adsorption is more pronounced under acidic conditions and in environments with low organic matter content. Meanwhile, certain cations in the soil, such as K^+^, can act as “bridges” to adsorb Cr(VI) onto the surface of soil minerals [39]. In contrast, anions such as SO_4_^2−^ and HPO_4_^2−^, which share structural similarities with CrO_4_^2−^, exhibit strong competitive adsorption and can significantly displace adsorbed Cr(VI), thereby promoting its remobilization.

These processes are interrelated and regulated by factors such as pH [40]. Under acidic conditions, increased H^+^ concentration promotes protonation of mineral surfaces, whereas under alkaline conditions, mineral adsorption capacity is reduced. Low redox potential enhances the stability and reducing capacity of iron and manganese-containing minerals. Multiple factors interact to collectively influence the ability of minerals to affect the migration and transformation of Cr(VI).

### 2.2. Soil Biota

Soil biota, as core drivers of soil ecosystems, comprises plants, animals, and microorganisms. These groups interact through material cycling, energy flow, and information exchange, collectively regulating the migration and transformation of hexavalent chromium (Cr(VI)) in soil [41].

#### 2.2.1. Soil Plants

Plants primarily absorb Cr(VI) via root systems. Cr(VI) enters root cells via anion transporters on the cell membrane and is subsequently transported through a series of physiological processes [42]. Root exudates such as organic acids chemically alter Cr(VI) speciation, which is directly in control of the Cr valence state. At the same time, microbial reaction from Cr(VI) reduction to Cr(III) in the rhizosphere is enhanced by the influence of community structure and activity of soil microorganisms [43].

Significant interspecific variation in Cr(VI) absorption capacity exists among plant species [44,45]. Phytoremediation utilizing diverse plant varieties has gained extensive research attention due to its cost-effective nature and high public acceptance for in situ Cr(VI) contamination remediation. However, Cr(VI) concentrations exceeding species-specific thresholds induce phytotoxicity. It was demonstrated in Vicia faba (faba bean) that Cr(VI) disrupts electron transport chains, induces chloroplast degradation, and impairs photosynthetic machinery [46]. The tolerance and uptake capacity of selected plant species to hexavalent chromium are summarized in Table 1 [47,48,49,50].

Concurrently, plant-mediated Cr(VI) reduction is modulated by environmental conditions. It reported that diminished soil reactive oxygen species (ROS) levels enhance plant uptake of water-soluble Cr(VI) species, adsorbed free state Cr(VI) fractions and Cr(VI) in chromate precipitates, while promoting chromium sequestration in metabolically inactive compartments such as cell walls and vacuoles [51].

#### 2.2.2. Soil Fauna

Soil fauna refers to heterotrophic animals that inhabit the soil environment and depend directly or indirectly on soil organic matter and microorganisms for survival; these include earthworms, nematodes, and others. Through activities such as burrowing, ingestion, and excretion, soil fauna alter the physical structure of the soil. These actions enhance soil porosity and permeability, thereby accelerating the migration of Cr(VI) [52,53]. The activity of earthworms, for example, promotes the decomposition of organic matter, increases the surface area of organic substrates, and facilitates the formation of humus and organic acids. Humic substances influence the migration and transformation processes of hexavalent chromium on mineral surfaces by providing functional-group binding sites, reducing the surface charge of minerals, and altering adsorption mechanisms. Simultaneously, humic substances promote the reduction of Cr(VI) to Cr(III) and form micelle-like structures with it, thereby enhancing the mobility and environmental persistence of Cr(VI) [54]. The intestinal environment of soil fauna is characterized by neutral pH, low redox potential, and high organic matter content. Mucus secretions contain reducing substances including cysteine and quinones, which can directly reduce Cr(VI) to Cr(III). Earthworms also secrete abundant humic acids, which enhance the solubility of heavy metals and nutrients in the soil while slightly lowering soil pH [55]. Investigations have also confirmed that earthworm-driven vermicomposting effectively reduces Cr(VI) toxicity and produces nutrient-rich agricultural fertilizers [56]. Additionally, earthworm activity stimulates microbial growth, further promoting Cr(VI) reduction.

The input of organic matter resulting from faunal activity provides a rich carbon source for microorganisms, enhancing their metabolic activity and consequently accelerating Cr(VI) reduction [57]. Faunal activities also improve soil structure, aeration, pH, and organic matter content. It should be noted that the effectiveness of soil fauna in mediating Cr(VI) migration and transformation varies under different pH conditions.

#### 2.2.3. Soil Microorganisms

Soil microorganisms play crucial roles in restoring soil health and quality, decomposing organic matter, and facilitating nutrient exchange between soil and plants [58]. Microorganisms interact with chromium ions through processes such as reduction, accumulation, adsorption, and precipitation. These interactions alter the geochemical fractionation of chromium, thereby influencing its bioavailability in the soil–plant system. Numerous microorganisms, such as *Aspergillus fumigatus*, *Rhizopus* sp., *Penicillium radicum*, and *Fusarium proliferatum* [59], interact with chromium ions through processes including reduction, accumulation, adsorption, and precipitation. These interactions alter the geochemical partitioning of chromium, thereby influencing its bioavailability within the soil–plant system. Microbial reduction of Cr(VI) proceeds primarily via enzymatic and non-enzymatic pathways [60,61]. Enzymatic reduction involves specific enzymes, such as chromate reductases, which transfer electrons to Cr(VI), reducing it to Cr(III). Non-enzymatic reduction relies on metabolic byproducts—such as organic acids and hydrogen—that chemically reduce Cr(VI).

Luo et al. [62] found that arbuscular mycorrhizal fungi enhance the production of arbutin, which is subsequently catalyzed by glucosidase and converted into hydroquinone. The hydroquinone then directly reacts with hexavalent chromium, reducing it to the less toxic trivalent chromium. The efficacy of Cr(VI) reduction is directly influenced by microbial activity. Under favorable environmental conditions that support microbial growth and reproduction, increased microbial activity enhances Cr(VI) reduction, leading to greater conversion of Cr(VI) to the less mobile Cr(III). It is found that variations in soil moisture and temperature significantly affect microbial biomass and enzyme activity [63]. Conversely, when soil environments are disturbed by pollution or other stressors, microbial activity may decline, weakening Cr(VI) reduction and potentially increasing its mobility.

## 3. Factors Affecting the Transport and Transformation of Cr(VI) in Groundwater

**Figure 3:**
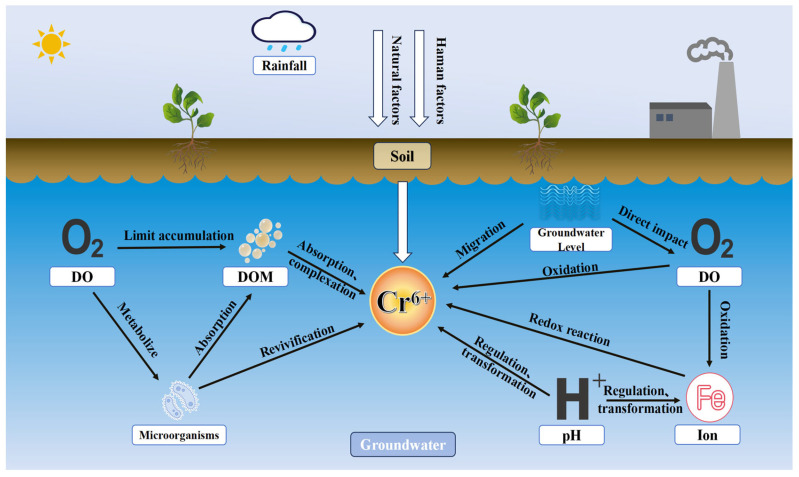
Schematic diagram of the influence of groundwater environmental factors on Cr(VI) migration and transformation.

Dissolved chromium in groundwater primarily exists as Cr(VI) and Cr(III). Its concentration depends on the chromium source, pH, dissolved oxygen (DO), coexisting anions, and hydraulic conditions (Figure 3). Groundwater with high chromium levels typically exhibits elevated pH, DO, bicarbonate (HCO_3_^−^), and sulfate (SO_4_^2−^). In aquifers containing exchangeable and silicate-bound chromium forms, groundwater flow rates are generally low. In bedrock aquifers rich in dissolved oxygen and with relatively low pH, oxidation of dissolved Cr(III) during silicate weathering leads to the formation of Cr(VI). Low flow rates contribute to the enrichment of Cr(VI) in such groundwater. In sediment aquifers, groundwater with high pH, high DO, and the presence of dissolved manganese facilitates the oxidation of adsorbed Cr(III). Following desorption of Cr(VI) from sediments, low flow rates promote the accumulation of dissolved Cr(VI) in groundwater [64].

### 3.1. Influence of Groundwater Physicochemical Properties

Compared to soil substrates, the groundwater system exhibits different hydrological and geochemical driving factors for the behavior of Cr(VI), primarily controlled by hydrodynamics, fluid dynamics, and spatial heterogeneity of redox gradients. Unlike the soil system, which interacts with solid phases, the migration of Cr(VI) in groundwater is governed by the chemical nature of the solution, where dissolved ions (including Ca^2+^, HCO_3_^−^, SO_4_^2−^) compete through ligand exchange and co-precipitation patterns to modulate the formation of Cr(VI) species. Additionally, the pH-dependent redox pair Cr(VI)/Cr(III) shows higher sensitivity to the electron flux from aqueous Fe^2+^ or dissolved organic matter. Furthermore, the distinctive mechanism of advection–dispersion plays a fundamental role in the transport of chromium ions, while the flow state of groundwater dictates the plume dispersion dynamics, promoting diffusion-limited transformation in low permeability aquifers, whereas high-flow systems favor the persistent oxidizing nature of Cr(VI), regulating the mechanisms.

#### 3.1.1. pH

The pH of groundwater is a critical factor governing Cr(VI) migration and transformation, exerting crucial control over key environmental chemical processes [65].

The pH of groundwater modulates Cr(VI) solubility; similar to its behavior in soil, the speciation and mobility of hexavalent chromium in groundwater varies under different pH conditions. pH also significantly influences redox reactions among groundwater ions, altering both the direction and kinetics of these reactions, which in turn modifies the valence state and chemical properties of Cr(VI). Furthermore, pH affects the adsorption behavior of Cr(VI) onto aquifer media by modifying solid–solution interactions, thereby impacting its transport and transformation. It demonstrated that Cr(III) exhibits low solubility, limited mobility, and reduced toxicity at pH > 5, contrasting with Cr(VI), which displays enhanced mobility and elevated toxicity in alkaline environments (pH > 6) [66]. Investigations have also confirmed that Cr(VI) was characterized by high pH dependence in redox conversion, which can be summarized by Fe(II)-mediated reduction efficiency decreased at pH 3–5 but increased at pH 6–8 [67].

In summary, groundwater pH profoundly influences Cr(VI) behavior through solubility control, redox regulation, and adsorption modulation [68]. Acidic conditions promote Cr(VI) reduction, diminishing toxicity and mobility, whereas alkaline conditions enhance migration potential and environmental dispersion risks.

#### 3.1.2. Dissolved Oxygen (DO)

Dissolved oxygen (DO) concentration refers to the amount of oxygen dissolved in the water column. In groundwater systems, DO primarily originates from aquifer sediments acting as non-reactive materials, with its distribution typically exhibiting a decreasing trend with depth. This pattern is governed by groundwater–atmosphere contact extent and geological structures [69].

Dissolved oxygen (DO) concentration exerts critical control over hexavalent chromium migration and transformation in groundwater systems [70], where oxidative conditions stabilize Cr(VI) while reductive environments facilitate its reduction to Cr(III). Fan et al. established a statistically significant positive correlation (r = 0.61, *p* < 0.05) between Cr(VI) levels and DO concentrations, confirming that Cr(VI) predominantly originates from Cr(III) oxidation wherein DO serves as a primary oxidant [71].

Concurrently, DO modulates microbial activity processes and ion-mediated transformations, as demonstrated by the finding that DO governs Fe(II)-driven Cr(VI) reduction kinetics at Fe(II): Cr(VI) = 3:1 stoichiometry ratio [67]. Under oxic conditions, DO promotes Cr(III) oxidation despite inherently slow reaction kinetics in groundwater, leading to progressive Cr(VI) enrichment. This occurs because prolonged residence times compensate for the slow reaction kinetics.

#### 3.1.3. Dissolved Organic Matter (DOM)

Dissolved organic matter (DOM) in groundwater constitutes a complex mixture enriched with reactive functional groups—including hydroxyl, carboxyl, phenolic hydroxyl, aromatic rings, and thiol moieties—which confer multifaceted chemical essential to hydrogeochemical processes. Substantial evidence confirms DOM’s significant influence on Cr(VI) migration and transformation through dual pathways, which can be described as adsorptive immobilization via complexation that restricts mobility and reductive transformation wherein electron-donating groups reduce toxic Cr(VI) to less mobile Cr(III) [72].

Investigations have also confirmed that during Cr(VI) reduction, amino and phenolic hydroxyl groups within DOM undergo concomitant oxidation to form aldehyde and carboxyl functionalities [73], thereby facilitating chromium valence transition while diminishing its bioavailability and environmental risk through oxidative state alteration.

Empirical observations reveal an inverse correlation between DOM concentration and Cr(VI) migration capacity in aquifer systems. Elevated DOM levels enhance both adsorptive sequestration through increased binding sites and thermodynamic favorability of reduction kinetics, collectively promoting Cr(VI) to Cr(III) conversion that effectively suppresses hexavalent chromium dispersion.

#### 3.1.4. Ionic Composition

The ionic composition of groundwater critically regulates Cr(VI) migration and transformation through mechanisms that vary with ion speciation. Calcium and magnesium ions precipitate with Cr(VI) to form low-solubility compounds, thereby reducing its mobility, while simultaneously catalyzing redox reactions that accelerate Cr(VI) transformation, as demonstrated in previous studies [67].

Sequential extraction analyses reveal that Cr(VI) derivation correlates with silicate weathering, where manganese oxides (MnO_2_) function as key redox mediators that oxidize Cr(III) to Cr(VI) through valence alteration [74]. Stratified redox conditions modulate these dynamics: in shallow aquifers where Mn oxide reductive dissolution occurs, Cr(VI) mobility becomes constrained, whereas in deep aquifers characterized by oxic–suboxic conditions, restricted Mn solubility enhances Cr(III) oxidation [75].

Ancillary ions exert supplementary influences, such as tetrahydroxyborate [B(OH)_4_]^−^ in coastal systems, which identified as inhibiting Cr(VI) reduction through competitive adsorption and solution property modification [76]. The influence of groundwater ion composition on Cr(VI) migration and transformation has multi-mechanism and multi-scale characteristics that depend upon ion speciation, concentration gradients, redox potential, and geological matrix properties (Table 2).

### 3.2. Influence of Hydrogeological Conditions

Although geological conditions vary significantly across regions, there are common influencing factors governing the migration and transformation of Cr(VI). Among these, groundwater flow velocity and water table fluctuations are the predominant factors controlling the vertical transport rate, as they modulate the mobility of Cr(VI) through advection–dispersion mechanisms.

#### 3.2.1. Groundwater Flow

Groundwater flow rates critically influence Cr(VI) migration and transformation, where the advective transport of directly dissolved Cr(VI) ions by groundwater functions as the predominant mechanism for their migration within aquifers. Elevated flow velocities extend dispersion distances and broaden contaminant dispersion ranges, as demonstrated through hydraulic gradient analysis along Baiyangdian Basin flow paths, which revealed progressive Cr(VI) enrichment correlated with decreasing flow rates due to intensified water–rock interactions during prolonged residence times [77]. This phenomenon is mathematically formalized by Wu et al. [78] using Fick’s law-advective diffusion principles:

(1) $$\begin{array}{l} \frac{\partial nC}{\partial t}=\frac{\partial }{\partial x}\left(nD_{xx}\frac{\partial C}{\partial x}+nD_{xy}\frac{\partial C}{\partial x}+nD_{xz}\frac{\partial C}{\partial x}\right)+\frac{\partial }{\partial y}\left(nD_{yx}\frac{\partial C}{\partial x}+nD_{yy}\frac{\partial C}{\partial y}+nD_{yz}\frac{\partial C}{\partial z}\right)\\ +\frac{\partial }{\partial z}\left(nD_{zx}\frac{\partial C}{\partial x}+nD_{zy}\frac{\partial C}{\partial y}+nD_{zz}\frac{\partial C}{\partial z}\right)-\frac{\partial nu_{x}C}{\partial x}-\frac{\partial nu_{y}C}{\partial y}-\frac{\partial nu_{z}C}{\partial z}+I \end{array}$$

Groundwater flow critically modulates Cr(VI) migration and transformation through hydrodynamic dispersion mechanisms, where the dispersion tensor components D_xx_, D_xy_, D_xz_, D_yx_, D_yy_, D_yz_, D_zx_, D_zy_, and D_zz_ (m^2^/s) characterize anisotropic diffusion; while C denotes contaminant concentration (mg/m^3^), u_x_, u_y_, and u_z_ represent directional seepage velocities (m/s), *n* signifies soil porosity, and I quantifies Cr(VI) source/sink terms from biogeochemical reactions (mg/m^3^·s), collectively validating flow as a primary migration driver.

Flow regimes indirectly regulate Cr(VI) transformation by redistributing contaminants and modifying geochemical milieus, where advective transport delivers upstream chemical signatures to downstream zones [79]. Oxygen-rich flows that introduce oxidizing conditions may cause the oxic environment into polluting area and thus inhibit Cr(VI) transporting progress to Cr(III), sustaining its low level of solubility, whereas flows conveying reductants from upstream regions can facilitate reductive precipitation to immobile Cr(III) hydroxides, reducing environmental risks through adsorption or precipitation [77].

Microbial Cr(VI) reduction is further mediated by flowing dynamics, which redistribute electron donors/acceptors to modulate metabolic activity of sulfate-reducing bacteria (SRB) and iron-reducing bacteria (IRB), while concurrently enabling exogenous reductant microbe inoculation that enhances in situ bioaugmentation potential.

Thus, groundwater flow velocity, directionality, and chemical composition establish an integrated regulatory 3D network wherein physical advection governs spatial contaminant dispersion [80], chemical conditions change in the chemical environment control the direction of redox reactions, and biological processes determine transformation kinetics and product stability.

#### 3.2.2. Groundwater Level Dynamics

Water table fluctuations modulate the migration and transformation of Cr(VI) by altering flow fields, redox conditions, and contaminant-media contact—a process that exhibits distinct seasonal characteristics and is driven by climatic cycles, precipitation intensity/frequency, and anthropogenic water extraction. Ma et al. [81] documented pronounced water table oscillations during the summer months under elevated temperatures, where shallow aquifers (<15 m depth) exhibit strong hydrologic connectivity to surface waters, enabling precipitation leaching and evaporative concentration that facilitate Cr(VI) enrichment and enhanced mobility. Conversely, deep aquifers (>50 m) display limited surface connectivity, low flow velocities (<0.01 m/day), and diminished hydraulic gradients, resulting in retarded Cr(VI) transportation [82].

Guo et al. [83] observed stratigraphic differentiation in Baiyangdian Basin aquifers, wherein deep aquifers (>150 m) exhibit elevated Cr concentrations under oxic–suboxic conditions, while shallow groundwater shows depressed Cr levels despite higher Fe/Mn concentrations indicative of reducing environments. Sequential extraction analyses reveal that Cr(VI) mobility in shallow zones is constrained by reductive dissolution of Mn oxides, whereas in deep aquifers, oxic conditions restrict Mn solubility, thereby enhancing Cr(III) oxidation to Cr(VI) and promotes Cr(VI) desorption.

Water table dynamics transcend simple dilution/concentration effects, establishing divergent Cr(VI) transformation mechanisms across depth gradients: shallow systems favor reductive immobilization through Mn/Fe cycling, while deep systems exhibit oxidative release characterized by slow hydrologic cycles that prolong contaminant residence times [84].

#### 3.2.3. Aquifer Media

The properties of different aquifer media vary depending on factors such as mineral composition, particle size, and specific surface area [85]. Permeability constitutes a pivotal physical parameter that modulates advective transport: highly permeable media with large, interconnected pores facilitate rapid groundwater flow, accelerating Cr(VI) migration, whereas low-permeability strata restrict flow velocities through constricted pore throats and poor connectivity, thereby impeding contaminant dispersion [86].

The particle size of aquifer media is closely related to its specific surface area [87], which significantly influences the migration of Cr(VI). Finer-grained media possess a larger specific surface area [77], providing more adsorption sites for Cr(VI) and retarding its migration. Studies have shown that, under certain conditions, the adsorption capacity of fine-grained media for Cr(VI) can reach significant levels, effectively reducing the concentration and migration rate of Cr(VI) in groundwater.

The chemical properties of aquifer media also affect the transport and transformation of Cr(VI). When the media contain iron oxides, these minerals carry a positive surface charge, while Cr(VI) typically exists in aqueous solution as oxyanions such as CrO_4_^2−^ and HCrO_4_^−^ [10]. The resulting electrostatic attraction facilitates the adsorption of Cr(VI) onto the surface of iron oxides. In addition, reactive functional groups like hydroxyl groups on the oxide surfaces can form stable complexes with Cr(VI) through chemical bonding, further enhancing adsorption and effectively reducing the migration ability of Cr(VI) in groundwater.

### 3.3. Microbial Mediation

Microorganisms constitute pivotal agents in Cr(VI) biogeochemical cycling within groundwater ecosystems, where their redox activities govern chromium speciation through phylogenetically diverse pathways [88]. Xia et al. [89] identified Proteo bacteria and Bacteroidetes as dominant phyla in Henan Province aquifers, which exhibit significant positive correlations (r > 0.75, *p* < 0.01) with Cr(VI) and SO_4_^2−^ concentrations, indicating chromium resistance and reduction capabilities.

Distinct toxicity responses emerge between microbial domains: Certain microorganisms, such as *Micrococcus* sp., directly reduce Cr(VI) to Cr(III) via extracellular enzymes or intracellular reductases, while others, such as Pseudomonas aeruginosa [90], mediate the adsorption of chromium onto cell surfaces or through secreted extracellular polymeric substances, or facilitate the formation of stable chromium precipitates, thereby limiting the mobility of hexavalent chromium [91]. Investigations have also confirmed that community-scale adaptations in Shanxi Loess Plateau aquifers, revealing significant divergence (*p* < 0.05) in microbial composition between low-Cr(VI) (<50 μg/L) and high-Cr(VI) (>50 μg/L) groundwater, with elevated diversity and richness under high Cr(VI) stress reflecting selective ecological pressure [71]. Environmental drivers, particularly pH and dissolved oxygen, which modulate these interactions, as evidenced by studies correlating microbial Cr(VI) reduction kinetics with geochemical gradients across redox transition zones [92].

## 4. Synergistic Effects in Soil–Groundwater Systems

**Figure 4:**
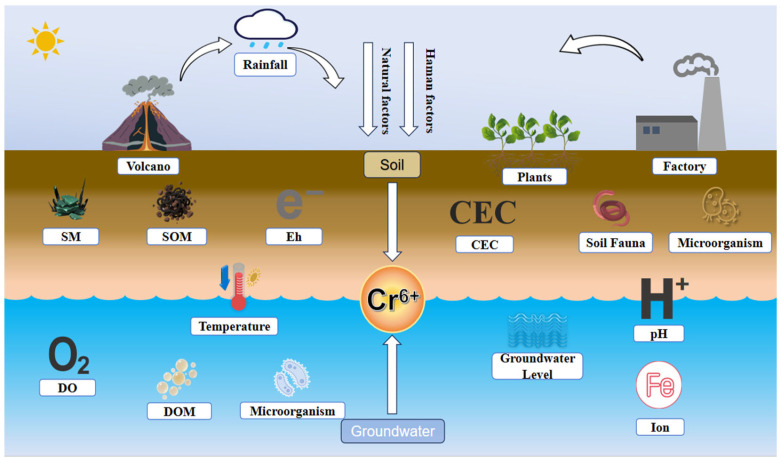
Factors influencing the migration and transformation of hexavalent chromium (Cr(VI)) in the soil–groundwater system.

The soil–groundwater continuum exhibits dynamic interdependence in Cr(VI) migration and transformation, wherein synergistic mechanisms transcend single-medium limitations. The core of this synergy lies at the soil–groundwater interface, particularly within the redox fluctuation zone influenced by water table dynamics (Figure 4). This zone is characterized by intense variations in hydrogeochemical parameters, active biological activity, and pronounced interfacial reactions, making it a hotspot for Cr(VI) valence transformation and speciation migration. It plays a decisive role in determining the environmental fate of Cr(VI). Furthermore, climate factors act as external drivers that indirectly influence Cr(VI) migration rates and speciation transformation. Temperature alters hydrodynamic conditions, reaction kinetics, and microbial activity, while precipitation regulates soil infiltration, groundwater recharge, and water level fluctuations. These processes collectively enhance the systemic synergistic effects. A holistic perspective is therefore essential to unravel the underlying mechanisms and support pollution control strategies.

### 4.1. Climatic Drivers

Climatic parameters include the encompassing temperature, monsoon-influenced precipitation regimes, seasonal humidity gradients, etc. Some parameters maintain Cr(VI) dynamics through interconnected pathways wherein precipitation governs the change in infiltration capacity and soil hydraulic conductivity, and these two factors directly impact the migration of Cr(VI) by controlling contaminant leaching in aquifers, while temperature modulates microbial metabolic rates and concurrently alters contaminant physicochemical properties, determining environmental persistence and transformation velocity rate.

#### 4.1.1. Temperature

Rising global temperatures intensify the urgency to elucidate thermal regulation mechanisms governing Cr(VI) behavior in soil–groundwater systems. Current studies indicate that temperature, as a key environmental factor, facilitates chromium migration more readily under elevated temperatures than under lower conditions. The oxidation of Cr(III) triggered by wildfires has been proposed as a significant pathway for the formation of Cr(VI) [93]. Furthermore, high temperatures can readily alter soil properties and composition, thereby indirectly influencing the speciation and mobility of chromium in soils. Zhao et al. [94] demonstrated through gradient thermal exposure experiments (40 °C, 120 °C, 500 °C) that the stability of Cr in soil remediated with FeSO_4_ under elevated temperatures (40 °C, 120 °C, and 500 °C). Treatments at relatively lower temperatures (40 °C and 120 °C) showed a tendency to further enhance stabilization efficiency, though no significant alteration in chromium speciation was observed. In contrast, under the relatively high temperature of 500 °C, the stabilization efficiency decreased significantly, resulting in increased leaching of both Cr(III) and Cr(VI).

Rising temperature significantly reduces the kinematic viscosity of water. A decrease in viscosity reduces the resistance to water flow through soil pores and groundwater aquifers, resulting in increased flow velocity. This, in turn, leads to accelerated transport of dissolved Cr(VI), enabling it to migrate faster and spread more widely. Additionally, elevated temperature generally follows the Arrhenius equation, markedly increasing the reaction rate at which Cr(VI) is reduced to the less toxic and less mobile trivalent form, Cr(III) [95].

Temperature is also one of the most critical environmental factors regulating microbial activity in soil and groundwater. Within a suitable range, higher temperatures significantly enhance microbial metabolic activity and growth rates. This directly results in a substantial increase in the rate of microbial-dependent Cr(VI) bioreduction [96]. Many dissimilatory metal-reducing bacteria (DMRB) as well as aerobic/anaerobic Cr(VI)-reducing bacteria exhibit higher activity at elevated temperatures [97]. Enhanced microbial activity also promotes the decomposition of organic matter, generating more reducing substances that indirectly facilitate chemical reduction.

Furthermore, increased temperature lowers the solubility of dissolved oxygen in water. Lower DO levels promote the formation of anaerobic or hypoxic conditions, thereby favoring reductive transformation [98]. Concurrent thermal acceleration of soil organic matter mineralization releases increased quantities of reductive functional groups and low-molecular-weight organic acids that promote Cr(VI) reduction, while simultaneously generating soluble dissolved organic matter (DOM) that may either complex with Cr(VI) or competitively occupy adsorption sites, potentially enhancing its mobility [87].

Collectively, elevated temperatures enhance dissolved Cr(VI) migration capacity through accelerated advection via viscosity reduction, intensified diffusion kinetics, and diminished adsorption affinity. Although thermal conditions substantially promote Cr(VI) reduction to Cr(III) via accelerated reaction kinetics and favorable anaerobic microenvironments, seasonal oscillations superimposed on climatic trajectories induce periodic variability in Cr(VI) transport rates and transformation efficiencies, thereby altering contaminant plume morphology, dispersion extent, and long-term environmental risks.

#### 4.1.2. Rainfall

Rainfall primarily regulates the migration and transformation of Cr(VI) in soil–groundwater systems by altering hydrological conditions. It can both enhance migration and promote transformation or immobilization. As the main driver for the vertical transport of Cr(VI) into deep soil and groundwater, rainwater carries dissolved Cr(VI) downward through soil pores [99]. Heavy rainfall can lead to the formation of preferential flow paths in the soil, allowing Cr(VI) to bypass the soil matrix and rapidly reach groundwater, significantly shortening the migration time and reducing opportunities for adsorption and degradation. When rainfall exceeds the soil’s infiltration capacity, surface runoff occurs, transporting soil particles adsorbed with Cr(VI) or dissolved Cr(VI) to downstream areas, thereby expanding the contamination scope [2].

Meanwhile, continuous rainfall recharges groundwater, causing a rise in the water table [84]. This submerges Cr(VI)-contaminated soil originally located in the unsaturated zone (vadose zone), transitioning it from an oxidative to a reducing environment, which may trigger the reduction of Cr(VI). The rising water level can also disturb sediments at the bottom of the aquifer, remobilizing previously adsorbed or precipitated Cr(VI) into flowing groundwater, leading to an expansion of the contamination plume or a rebound in concentration.

Rainwater introduces dissolved oxygen, which may temporarily increase the Eh in local microenvironments within the soil, hindering Cr(VI) reduction. However, as infiltrating water consumes the dissolved oxygen and the submerged soil forms an anaerobic environment, Eh decreases significantly, creating conditions favorable for both chemical and biological reduction of Cr(VI) [100]. The pH may also change; if rainfall has low pH, it can acidify the soil solution. Under acidic conditions, Cr(VI) primarily exists as HCrO_4_^−^, which exhibits different adsorption behavior compared to CrO_4_^2−^. Acidic conditions generally inhibit the adsorption of anionic Cr(VI) onto positively charged soil minerals and may even cause desorption of previously adsorbed Cr(VI). Although low pH favors chemical reduction, excessively acidic conditions can inhibit microbial activity, thereby impeding bioreduction.

Rainfall provides essential moisture for microbial activity. Precipitation after drought periods can rapidly activate dormant Cr(VI)-reducing bacteria, significantly enhancing the bioreduction rate. Rainwater also dissolves and transports organic matter, nitrate, sulfate, and other substances downward, supplying microorganisms with carbon, nitrogen, and sulfur sources, as well as electron donors necessary for Cr(VI) reduction, thereby promoting bioreduction processes. Alternating wet–dry cycles can markedly alter the structure and activity of soil microbial communities, influencing their capacity to reduce Cr(VI) [97].

### 4.2. Migration, Transformation, and Comprehensive Impact of Cr(VI) at the Soil–Groundwater Interface

The soil–groundwater interface, situated within the critical transition zone between vadose and saturated zones, functions as a pivotal domain for Cr(VI) phase transfer and speciation shifts, where water table dynamics drive saturation–desaturation cycles that significantly modulate biogeochemical interactions [101]. Under hydrodynamic conditions, dissolved Cr(VI) migrates primarily through advection and diffusion, with its direction and rate of transport influenced by the pressure gradient and hydraulic conductivity. Adsorbed Cr(VI), in contrast, exhibits retarded mobility due to attachment to particle surfaces, while vapor-phase diffusion in unsaturated regions further influences redox equilibria and adsorption–desorption dynamics. Using a three-dimensional experimental apparatus, Zhao et al. [102] demonstrated that Cr(VI) undergoes bidirectional migration, both vertical and horizontal, under groundwater flow driving forces. Field studies have further confirmed that vadose zone lithology and thickness are key factors controlling chromium migration, with a weaker inhibitory effect on Cr(VI) compared to Cr(III), and that Cr(VI) can persist over extended periods in groundwater.

The soil–groundwater interface, representing a characteristic water-table fluctuation zone, exhibits periodic cyclic transitions between saturated and unsaturated states. Throughout water-level dynamics, soil particles undergo repeated saturation–release-phase changes. Within the groundwater table fluctuation zone (GTF), fluctuating redox conditions in the vadose zone facilitate the oxidation of Cr(III). Human activities such as groundwater extraction and agricultural irrigation, along with nutrient availability, further enhance the generation of Cr(VI).

The transport of Cr(VI) in unsaturated soils and saturated aquifers is governed by multiple coordination mechanism. Primarily, chromium can undergo long-distance migration via advective transport in groundwater, with its velocity showing a linear correlation with the Darcy flux. Secondly, dispersion, which is comprise both mechanical dispersion and molecular diffusion constituting another major factor influencing Cr(VI) mobility. Using isotopic tracers, Wei et al. [103] demonstrated that transverse dispersion contributes to the dilution of Cr(VI) within flowing groundwater, resulting in the formation of a contaminant plume. Ultimately, co-transport of soil colloids and contaminants represents a critical mechanism facilitating the migration of chromium from the unsaturated zone into saturated aquifers. Given its high specific surface area and surface energy, chromium readily adsorbs onto colloidal surfaces, thereby undergoing colloid-facilitated transport.

The dynamic environmental characteristics and coupled multi-process interactions at the soil–groundwater interface determine the complexity and nonlinear behavior of Cr(VI) transport and transformation. Future studies should integrate high-resolution monitoring techniques with multiphase flow reactive transport models to quantify the synergistic effects of water table fluctuations, human activities, and colloidal behavior on the long-term fate of Cr(VI). Furthermore, there is a need to develop contamination control strategies based on the regulation of interfacial conditions, thereby providing theoretical support for the adaptive management of chromium-contaminated sites under warming conditions.

As a highly mobile and toxic heavy metal pollutant, hexavalent chromium (Cr(VI)) exerts substantial impacts on the soil–groundwater system through complex migration–transformation processes and associated environmental and ecological effects. Upon entry, the transport and environmental fate of Cr(VI) in soils are influenced by multiple factors, including external forces and intrinsic soil properties such as pH, organic matter content, and mineral composition, as well as the inherent physicochemical characteristics of Cr(VI). Adsorption of Cr(VI) onto soil organic matter and minerals occurs via electrostatic attraction, ligand exchange, and surface complexation [104]. The rich functional groups in organic matter and exchangeable cations on mineral surfaces actively interact with Cr(VI). Subsequent redox reactions may consume organic matter or modify the surface charge, thereby affecting soil fertility and buffering capacity.

Beyond direct impacts on the soil–groundwater system, Cr(VI) poses considerable ecological risks. Invertebrates such as earthworms ingest Cr(VI) during burrowing and feeding, leading to internalized inhibition of mitochondrial function and disruption of metabolic processes [105]. Plant roots absorb Cr(VI) through both active and passive mechanisms, leading to its accumulation in root tissues. Although this may partially limit further translocation, Cr(VI) induces strong phytotoxicity by disrupting cell membrane integrity, interfering with nutrient uptake, inhibiting photosynthetic enzymes, impairing water balance, and provoking excessive reactive oxygen species (ROS). The accumulation of reactive oxygen species causes oxidative stress, resulting in suppressed seed germination, reduced biomass, chlorosis, necrosis, and even plant death [75]. Long-term Cr (VI) exposure also reduces the diversity and activity of microbial communities in the soil–groundwater system, decreasing the secretion of degradative enzymes. However, certain microorganisms can utilize Cr (VI) as an electron acceptor in their metabolic processes, contributing to its reduction and degradation to some extent [106].

The dynamic environmental attributes and coupled multi-process interactions at the SGI determine the complexity and nonlinear behavior of Cr(VI) transport and transformation. Future studies should integrate high-resolution monitoring techniques with multiphase flow reactive transport models to quantify the synergistic effects of water table fluctuations, human activities, and colloidal behavior on the long-term fate of Cr(VI). There is also a need to develop contamination control strategies based on the regulation of interfacial conditions, thereby providing theoretical support for adaptive management of chromium-contaminated sites under warming conditions.

## 5. Effects of Coexisting Pollutants on the Migration and Transformation of Cr(VI)

### 5.1. Microplastics

Microplastics act as environmental vectors for heavy metals and exhibit significant adsorption potential for pollutants such as chromium. They readily coexist with Cr(VI) in both aquatic and terrestrial environments, forming co-contamination that subsequently impacts aquatic organisms and crop growth, leading to the accumulation of heavy metals within living organisms. A meta-analysis by Bi et al. [107] indicated that most microplastics demonstrate strong adsorption affinity for heavy metals, with polyethylene and polyvinyl chloride showing particularly notable adsorption efficacy for chromium; this phenomenon was further corroborated by the research of Godoy et al. [108]. The adsorption behavior of microplastics is jointly influenced by their inherent properties and the speciation of heavy metals, underscoring their pivotal role as carriers within co-contamination systems.

Beyond adsorption, microplastics can actively alter the speciation and migration behavior of Cr(VI) through surface photochemical processes. Polystyrene microplastics generate reactive oxygen species under light irradiation, reducing the highly mobile and toxic Cr(VI) to the less toxic, readily precipitable Cr(III). This transformation has been verified in real water bodies, where it mitigates the toxicity of Cr(VI) to Lemna minor and Daphnia magna [109]. These findings indicate that microplastics are not merely passive carriers of heavy metals but can actively regulate their environmental fate and associated ecological risks through physicochemical synergies. Therefore, the interactive effects between microplastics and heavy metals warrant significant attention in the assessment of co-contamination.

### 5.2. Pesticides

Certain pesticides, such as chlorpyrifos [110], contain oxygen- and nitrogen-bearing functional groups (e.g., amino and carbonyl groups) that can act as electron donors and form complexes with Cr(VI). Their degradation products may compete with chromate ions for adsorption sites on soil particle surfaces, thereby reducing the immobilization of chromium in soils. Through field-based toxicity experiments on heavy metals and pesticides, Fang et al. [111] concluded that pesticides and Cr(VI) can form new complexes via surface complexation, altering their mobility and toxicity. In contrast, a study by Yu et al. [112] indicated that the effect of chromium on pesticides is pronounced, whereas the influence of pesticides on chromium is relatively minor. The addition of Cr(VI) significantly prolonged the degradation half-life of imidacloprid in soil, while the addition of imidacloprid had little effect on the natural aging processes (adsorption, reduction, and fixation) of Cr(VI), with only slight variations in the reduction rate of Cr(VI) across different soils.

In co-contamination systems, pesticides primarily promote the desorption and migration of Cr(VI) through competitive adsorption and may indirectly interfere with its valence transformation via chemical complexation. Although the direct impact may be limited, as a key anthropogenic driver, pesticide application indirectly regulates the environmental behavior of Cr(VI) by altering soil properties. Therefore, the synergistic effects of coexisting pesticides should also be considered when assessing the contamination risk and remediation strategies for Cr(VI).

### 5.3. Surfactants

Surfactants are a class of synthetic organic compounds widely used in industrial production and household applications due to their properties of solubilization and cleansing [113]. A primary pathway for surfactants to enter soil is through acid mine drainage generated during mineral flotation processes. If these wastewater streams containing surfactants are not properly treated, they can infiltrate the soil, altering its mineral composition and heavy metal distribution. Once in the soil, surfactants can interact with iron oxides and other environmental contaminants, thereby affecting interfacial processes and soil stability [114]. The presence of these surfactants in the environment can lead to significant ecological issues. In aquatic ecosystems, foaming caused by surfactants reduces oxygen levels and degrades water quality [113].

A study by Chen et al. [115] revealed that the type of surfactant and environmental pH jointly regulate the transformation pathways of chromium-bearing schwertmannite (Sch-Cr), thereby profoundly influencing the migration and fate of hexavalent chromium. Under acidic conditions, the anionic surfactant SDBS promotes mineral transformation to goethite and competes for adsorption sites, leading to the release of chromium in dissolved forms and increasing environmental risks. In contrast, the cationic surfactant CTAB inhibits mineral transformation, enhances surface positive charge, and promotes chromium fixation within newly formed minerals. Under neutral conditions, both surfactants inhibit the formation of hematite; however, SDBS still facilitates chromium release through micelle effects, while CTAB continues to enhance chromium immobilization. This indicates that the widespread presence of surfactants can significantly alter the mobility and ecological toxicity of chromium in soil and aquatic environments.

## 6. Conclusions and Prospects

Hexavalent chromium (Cr(VI)), as a toxic heavy metal, poses significant risks to both human health and ecosystem safety. Understanding its transport and transformation behavior in environmental media is therefore essential for effective pollution control. This study systematically analyzes the influence of soil and groundwater interactions on the behavior of Cr(VI), along with the specific mechanisms of relevant contributing factors.

In the soil environment, the speciation, solubility, and mobility of hexavalent chromium are co-regulated by physicochemical properties—such as pH, redox potential (Eh), cation exchange capacity (CEC), soil organic matter (SOM), and soil minerals—as well as biological factors including plants, animals, and microorganisms. In groundwater systems, the migration and transformation of Cr(VI) are primarily controlled by physicochemical parameters like pH, dissolved oxygen (DO), dissolved organic matter (DOM), and ionic composition; hydrogeological conditions such as flow velocity, water level dynamics, and aquifer media; as well as microbial communities possessing Cr(VI) resistance and reduction capabilities. Under coupled soil–groundwater conditions, climatic factors—where temperature influences hydrodynamics, reaction kinetics, and microbial activity, and precipitation alters hydrological conditions affecting Cr(VI) transport and reduction—along with the soil–groundwater interface, serve as core influencing factors. Moreover, the factors within this integrated system exhibit coupled interactions, differing notably from the regulatory mechanisms in single-medium environments. The result indicates that the migration and transformation of Cr(VI) are co-determined by physicochemical properties of soils and groundwater, as well as biologically mediated processes. These factors do not act in isolation but interact synergistically, collectively influencing the speciation and mobility of Cr(VI). The soil–groundwater interface, serving as a transitional zone between different phases within the system, represents a critical domain where key migration and transformation processes occur.

This study provides a theoretical framework for hexavalent chromium (Cr(VI)) contamination remediation. To advance the understanding of Cr(VI) environmental behavior across multiple media and support effective pollution prevention and remediation, future research should prioritize the following directions:

(1) Mechanistic investigation of multi-factor synergies is urgently needed to deepen the understanding of cooperative mechanisms among physicochemical properties and biological processes during Cr(VI) transport. The behavior of Cr(VI) in coupled water–soil systems is influenced by complex interacting factors. Developing conceptual and numerical models that simulate Cr(VI) transport under varying factor combinations will help quantify the strength and direction of these interactions, providing a theoretical basis for accurate prediction of Cr(VI) dynamics.
(2) Comprehensive indicator development integrating hydro-pedological and anthropogenic factors should be advanced through frameworks that incorporate biological activity and human impacts into a holistic indicator system, wherein biological parameters such as microbial community structure and enzyme activity, which can help assess the role of biotransformation in Cr(VI) mobility. At the same time, human activities including industrial emission intensity and agricultural fertilizer use, should be quantified to evaluate their influence on Cr(VI) levels in soil and groundwater.
(3) Conducting quantitative mechanistic studies on the migration and transformation of hexavalent chromium (Cr(VI)) enables more effective intervention in its transport pathways. By elucidating the influence mechanisms of various factors on Cr(VI) mobility, targeted measures can be implemented to control its concentration at the source within the water–soil coupled system. For already contaminated water–soil systems, quantitative mechanistic research provides scientific guidance for remediation. By simulating and predicting the effects of restoration strategies on Cr(VI) transport, such studies help enhance remediation efficiency and support the sustainable management of water and soil environments.
(4) Conduct multimedia toxicity tests for Cr(VI) to establish quantitative relationships among environmental concentrations, bioavailability, and toxic effects. Based on risk assessment models incorporating toxicological endpoints, link the environmental behavior of Cr(VI) directly to human health and ecological risks, thereby promoting the application of toxicological evidence in environmental remediation technologies.

## Figures and Tables

**Table 1:** Summary of tolerance and uptake capacity of selected plant species to hexavalent chromium.

Types of Plants	Mechanism
*Brassica juncea*	*Brassica juncea* (Indian mustard) typically exhibits bioaccumulation and translocation factors greater than 1 for hexavalent chromium, enabling it to absorb and transfer Cr(VI) to its aboveground tissues. Combined application with plant growth-promoting rhizobacteria and earthworms significantly enhances its uptake and tolerance, demonstrating its utility in remediating soils in mining areas. This integrated approach can upregulate the expression of metal transporter genes, directly promoting chromium absorption.
*Triticum aestivum*	*Triticum aestivum* is relatively sensitive to chromium(VI), exhibiting a low no observed effect concentration (NOEC) for growth, and thus can serve as a sensitive indicator plant for chromium contamination in ecological risk assessment.
*Zea mays* L.	*Zea mays* L. is a hyperaccumulator plant suitable for the phytoremediation of Cr(VI)-contaminated soils. It is also widely cultivated and readily adopted by farmers. Therefore, maize can be prioritized as a hyperaccumulator for chromium remediation of agricultural soils contaminated with Cr(VI).
*Vetiveria zizanioides*	*Vetiveria zizanioides* can sequester and immobilize large amounts of hexavalent chromium within its root tissues, with minimal translocation to aerial parts. However, its tolerance to high concentrations of chromium is limited, and elevated levels can still lead to a reduction in biomass.

**Table 2:** The influence of various ions on hexavalent chromium and its mechanism.

Ion	Mechanism	Impact on Cr(VI)
Ca^2+^	CrO_4_^2−^ can directly form calcium chromate precipitate with Ca^2+^; owing to its similar ionic radius and structure to CO_3_^2−^, it can also incorporate into the calcite (CaCO_3_) lattice through doping.	It facilitates the formation of Cr(VI)-bearing calcium carbonate minerals, thereby immobilizing Cr(VI), while carbonation treatment can release and extract Cr(VI).
Mn(II)/MnO_2_	It can directly oxidize relatively stable trivalent chromium (Cr(III)) in the environment into hexavalent chromium (Cr(VI)) with higher mobility; meanwhile, reductive dissolution of manganese oxides occurs during this process, which consequently restricts the migration capacity of Cr(VI).	It promotes the formation of Cr(VI) and regulates its migration.
[B(OH)_4_]^−^	As a buffering agent, B(OH)_4_^−^ can regulate or stabilize the aqueous environment in groundwater. Furthermore, it competes with Cr(VI) for adsorption sites on sediment or other carrier surfaces, thereby hindering the adsorption and immobilization of Cr(VI).	It significantly inhibits the reduction of Cr(VI) to Cr(III).
Fe(III)	Under acidic conditions, Fe(III) provides adsorption sites for Cr(VI) and facilitates the oxidation of Cr(III) by H_2_O_2_/HO. In contrast, under alkaline conditions, Fe(III) competitively consumes H_2_O_2_.	Under acidic conditions, it promotes the re-oxidation of Cr(III), whereas under alkaline conditions, it suppresses the oxidation of Cr(III).

## Data Availability

The data presented in this study are available upon request from the corresponding author.
